# Prevalence and associated factors of condomless receptive anal intercourse with male clients among transgender women sex workers in Shenyang, China

**DOI:** 10.7448/IAS.19.3.20800

**Published:** 2016-07-17

**Authors:** Yong Cai, Zixin Wang, Joseph TF Lau, Jinghua Li, Tiecheng Ma, Yan Liu

**Affiliations:** 1 Department of Community Health and Family Medicine, School of Public Health, Shanghai Jiao Tong University, Shanghai, China; 2 Centre for Health Behaviours Research, The Jockey Club School of Public Health and Primary Care, The Chinese University of Hong Kong, Hong Kong, China; 3 Shenzhen Research Institute, The Chinese University of Hong Kong, Shenzhen, China; 4 Shenyang Consultation Centre of AIDS Aid and Health Service, Shenyang, China

**Keywords:** transgender women sex workers, unprotected anal intercourse, associated factors, HIV infection, China

## Abstract

**Introduction:**

Globally, transgender women sex workers have a high prevalence of HIV and condomless receptive anal intercourse with male clients (CRAIMC). We investigated the prevalence of CRAIMC and factors associated with CRAIMC among transgender women sex workers in China.

**Methods:**

In 2014, we anonymously interviewed 220 transgender women sex workers face to face in Shenyang, China. Those who self-reported as HIV negative or as having unknown HIV serostatus were invited to take up free, anonymous HIV rapid testing (*n=*183); 90 did so. Using CRAIMC in the last month as the dependent variable, three types of associated factors were investigated, in addition to background factors: feminizing medical interventions, sex work and perceptions related to condom use. Univariate and multiple logistic regression models were fitted.

**Results:**

Of the participants, 16.8% self-reported as HIV positive and 9.1% were detected to be HIV positive through free HIV testing; 26.8% had had CRAIMC in the last month, 45.5% had performed sex work in other Chinese cities (last year), and 23.2% had had condomless anal intercourse with men who were non-clients. In the adjusted analysis, significant factors associated with CRAIMC (last month) included the following: 1) any feminizing medical intervention performed (adjusted odds ratio, AOR: 2.22); 2) sex-work-related factors, including recruitment of male clients most often at hotels (AOR: 5.02) and charge per episode of transactional sex (201 to 400 RMB, AOR: 0.27; reference group: ≤100 RMB); and 3) perceptions related to condom use, including perceived transgender identity's impact on condomless sex such as wearing feminine attire, concern about exposing their status as a transgender woman to male clients (AOR: 1.20) and perceived self-efficacy of consistent condom use with male clients (AOR: 0.56). Perceived self-efficacy of consistent condom use with male clients fully mediated the association between perceived transgender identity's impact on condomless sex and CRAIMC.

**Conclusions:**

HIV prevalence among transgender women sex workers was high but probably underestimated. The high prevalence of condomless anal intercourse with male non-clients and high mobility in sex work among this population in China are causes for concern. Risk factors for CRAIMC were multidimensional and should be considered when designing interventions targeting transgender women sex workers. Such interventions are urgently needed.

## Introduction

Transgender women are assigned male at birth and currently identify as female/transgender/transsexual women [1–3]. They may express their gender identity *via* interventions (e.g. cosmetic surgery, hormone treatment and sex reassignment surgery) and/or specific behaviours (e.g. wearing feminine attire) [1]. In most countries, it is difficult or impossible for transgender women to change their legal gender identification, creating difficulties for them in accessing healthcare, education and employment [3,4]. The percentage of transgender women engaged in sex work has been estimated at 24 to 75% in the United States [5], 9% in Singapore [6], 58.8% in Brazil [7], 61.3% in Thailand [8], 66.7% in Uruguay [9] and 54 to 80% in Asia [3].

Transgender women form a key population that requires focused attention for HIV prevention [10]. Meta-analyses have reported an HIV prevalence of 19.1% among transgender women (49 times higher than that of the general adult population) and 27.3% among transgender women sex workers [10,11]. Other studies of transgender women sex workers reported an HIV prevalence of 66.7% in India and 67.9% in the United States, which was higher than that of male (15.1%) and female (4.3%) sex workers as reported by a meta-analysis covering 14 countries [11]. Despite the large size of the population of transgender men and women in mainland China, estimated at 400,000 [12], only one survey (*n=*52) has recorded self-reported HIV prevalence (11.1%) [13], and no data is available for transgender women sex workers.

Previous studies have reported high prevalence of condomless receptive anal intercourse with male clients (CRAIMC) among transgender women sex workers: 12 to 77% in the United States [5], 25% in South Africa [14], 26.9% in Thailand [1], 29.8% in Italy [15] and 92% in Pakistan [16]. We found only two quantitative studies that reported risk factors (e.g. financial pressure) and protective factors (e.g. subjective norms) associated with CRAIMC [1,17], and three qualitative studies that described reasons for practising CRAIMC [18–20]. These studies were conducted outside China.

Perceptions related to condom use have been associated with condomless sex with clients among male and female sex workers [17,21]. Although theory-based interventions have been found to be more effective than non-theory-based ones [22], behavioural health theories have not been utilized to study factors associated with CRAIMC among transgender women sex workers. The health belief model (HBM) [23] has been used to explain risk behaviours among sex workers more generally [21,24] and was used in this study. The HBM postulates that perceived susceptibility, perceived severity, perceived benefits, perceived barriers, cues to action and perceived self-efficacy are determinants of health-related behaviours [17,21,24].

We investigated four types of factors that may be associated with CRAIMC: 1) background variables (sociodemographics, HIV serostatus and anal intercourse with non-commercial male sex partners), 2) feminizing medical interventions (cosmetic surgery, hormone treatment and sexual reassignment surgery), 3) variables related to sex work (e.g. venue, charge and sex work in other cities) and 4) perceptions related to condom use (both perceived transgender identity's impact on condomless sex and perceptions related to condom use derived from the HBM). We further tested two hypotheses: 1) self-efficacy for condom use with male clients would mediate the association between feminizing medical interventions and CRAIMC and 2) self-efficacy for condom use with male clients would mediate the association between perceived transgender identity's impact on condomless sex and CRAIMC.

## Methods

### Study design

A cross-sectional study was conducted in Shenyang, China, from April to July 2014. Inclusion criteria were as follows: 1) age ≥18 years, 2) self-identification as a transgender woman, 3) anal intercourse with ≥1 male client (last three months) and 4) wearing feminine attire and make-up during sexual practice with men. In the absence of a sampling frame, it was not feasible to perform probability sampling. Instead, the staff of a non-governmental organization (NGO) who provided services to transgender women sex workers contacted all their clients and performed outreach for recruitment. Referrals were also made by some participants. Prospective participants were assured that refusing to participate in the study would not affect their right to use any services and that they could quit at any time without being questioned. A total of 282 eligible transgender women sex workers were approached for the study; 62 declined to participate in the study and 220 (78%) provided written informed consent and completed an anonymous face-to-face interview in settings with privacy ensured.

In the survey, 37 participants self-reported being HIV positive. The 183 who self-reported being HIV negative or having an unknown HIV serostatus were invited to take a finger-prick HIV rapid test (Alere Determine™ HIV-1/2 rapid HIV screening test, Alere Inc, Waltham (MA), United States; sensitivity=99.75%; specificity=100%); 49.2% (90/183) agreed. Those who tested HIV negative were given post-test counselling. Those who showed HIV-positive test results received psychological support and underwent confirmatory testing at the local Center for Disease Control and Prevention (CDC). All were subsequently confirmed as HIV positive and referred to appropriate services. Eight of them subsequently started antiretroviral treatment. Monetary compensation (50 RMB or 8.1 USD) was given to participants upon completion of the procedures. Ethics approval was obtained from the Survey and Behavioural Research Ethics Committee of the Chinese University of Hong Kong.

### Measures

#### Participant profiles

Information collected included sociodemographics (age, education level, monthly personal income, city of residence (*hukou*) and duration of stay in Shenyang), utilization of any HIV prevention services in the last six months, any CRAIMC in the last month, any anal intercourse with male sex partners who were non-clients in the last month and, if affirmative, whether condomless anal intercourse was involved. HIV status (self-reported as HIV positive, tested HIV positive, tested HIV negative and self-reported as HIV negative or unknown HIV status but refused testing) was recorded (Table 1).

**Table 1:** Background characteristics of transgender sex workers

	%
**Sociodemographics**	
Age group	
25 or less	23.6
>25 to 30	27.3
>30 to 40	39.1
>40	10.0
Education level	
Primary or below	11.9
Junior secondary	35.9
Senior secondary	41.4
Tertiary	10.9
Monthly income (in RMB)^a^	
3000 or less	25.9
3001 to 5000	32.3
5001 to 8000	18.2
>8000	23.6
Residents of Shenyang (*hukou*)	
Yes	12.7
No	87.3
Duration of stay in Shenyang	
≤1 year	18.2
1 to 5 years	37.7
>5 years	44.1
HIV prevention services utilized in the last six months	
No	8.2
Yes	91.8
**HIV serostatus**	
Self-reported as being HIV positive	16.8
Tested as positive in rapid HIV testing	9.1
Tested as negative in rapid HIV testing	31.8
Self-reported HIV negative or having unknown HIV serostatus but refused to take up HIV testing	42.2
**Sexual behaviour in the last month**	
Had condomless receptive anal intercourse with any male clients	
No	73.2
Yes	26.8
Had anal intercourse with non-commercial male sex partner(s)	
No	11.4
Yes	88.6
Had condomless anal intercourse with non-commercial male sex partner(s) (among those having non-commercial male sex partners, *n=*195)	
No	73.8
Yes	26.2
**Variables related to feminizing medical interventions (yes, %)**	
Cosmetic surgery	13.2
Hormone treatment	2.3
Sex reassignment surgery to remove testes, penis and scrotum	0.5
Sex reassignment surgery to reconstruct female genital	0.5
Any of the above	15.0

*Note*: *n*=220

a 1 USD=6.2 RMB at the time of survey.

RMB, renminbi; USD, US dollars.

#### Feminizing medical interventions

Participants were asked whether they had taken up cosmetic surgery, hormone treatment and/or undergone sex reassignment surgeries (removal of testes, penis and scrotum and surgeries for reconstruction of female genitalia) (Table 2).

**Table 2:** Sexual practices with clients

	%
Channels most often used to recruit clients	
Hotel	14.5
Park	44.1
Internet	41.1
Venues where sexual practices with clients took place	
Hotel	24.5
Rented room	45.0
Client's home	2.7
Park	27.7
Engaged in sexual practices with clients in other Chinese cities in the last year	
No	54.5
Yes	45.5
Engaged in sexual practices with clients in other countries in the last year	
No	96.8
Yes	3.2
Number of male sex clients in the last week	
1 to 10	65.4
11 to 20	19.1
21 to 30	6.4
>30	9.1
Number of female sex clients in the last week	
0	95.5
1 to 10	4.5
Average charge per episode of sex with clients (in RMB)^a^	
≤100 RMB	35.0
101 to 200 RMB	15.0
201 to 400 RMB	19.1
≥401 RMB	20.9
Ever used alcohol prior to or during sex with clients in the last month	
No	77.7
Yes	22.3
Ever used drugs prior to or during sex with clients in the last month	
No	79.1
Yes	20.9
Estimated proportion of male clients that might be aware of one's transgender women status during sex with clients	
All/majority	28.6
Minority/none	47.7
Uncertain	23.6

*Note: n=*220

a 1 USD=6.2 RMB at the time of survey.

RMB, renminbi; USD, US dollars.

#### Variables related to sexual practice with clients

Participants were asked about the channel that was most frequently used to recruit sex clients, venues where sexual intercourse with clients took place, the average charge per episode of sex with male clients, the number of male and female clients in the last week, drug/alcohol use prior to or during sex with male clients, any performance of sex work in other Chinese cities/other countries and the proportion of their male clients that might be aware of their identity as transgender women (Table 2).

#### Perceptions related to HIV and condom use

Three scales were constructed for this study and were based on the HBM: 1) the four-item Perceived Susceptibility for HIV Scale, 2) the six-item Perceived Barrier against Condom Use Scale and 3) the three-item Perceived Self-efficacy of Consistent Condom Use Scale (response categories: 1=strongly disagree, 5=strongly agree). In addition, participants were asked to assess their transgender identity's impact on condomless sex (i.e. whether wearing feminine attire during sexual practice with clients, worrying about clients discovering their gender identity, avoiding exposing their genitals to male clients and avoiding talking to male clients would increase risk of CRAIMC). The four-item Transgender Identity's Impact on Condomless Sex Scale was then constructed by summing up responses to these four items. Another item, “reminders for condom use were displayed in the venues where sexual practice with clients took place” was asked to assess cue to action. All the scale items are listed in Table 3.

**Table 3:** Scales related to HIV and condom use

	%	Mean scale score (SD)
**Perceived susceptibility to HIV transmission** (high/very high, %)		
Perceived risk of HIV infection *via* sexual intercourse with male sex partners.	24.5	
Perceived risk of HIV infection *via* sexual intercourse with clients.	39.5	
Perceived risk of transmitting HIV to non-commercial male sex partners.	26.9	
Perceived risk of transmitting HIV to male clients.	22.8	
*Perceived susceptibility for HIV scale*		8.8 (2.8)
**Perceived barriers to condom use** (agree/strongly agree, %)		
Condom was not available in venues for sexual practice with clients.	27.3	
It is inconvenient for me to use condoms with clients.	5.5	
I worry about police arrest when carrying condoms with me.	30.9	
Many male clients would not want to use condoms.	39.1	
Condom use with male clients would reduce my income.	39.1	
Condom use with male clients would shorten the duration of sexual intercourse.	39.4	
*Perceived barriers against condom use scale*		15.6 (4.2)
**Perceived cue to action for condom use** (agree/strongly agree, %)		
Reminders for condom use were displayed in the venues where sexual practices with clients took place.	50.0	
**Perceived self-efficacy of consistent condom use** (agree/strongly agree, %)		
I can suggest to clients that they use condoms.	98.2	
I can persuade clients who do not want to use condoms to use condoms.	71.8	
I am confident in using condoms during all episodes of sexual practices with clients.	78.1	
*Perceived self-efficacy in consistent condom use scale*		11.3 (2.1)
**Transgender identity's impact on condomless sex** (agree/strongly agree that circumstance would increase risk of CRAIMC, %)		
Wearing feminine attire during sexual practice with male clients.	18.7	
Worry that male sex clients might know about your identity as a transgender women.	10.0	
Attempts to avoid exposing your genitalia to male clients.	13.2	
Avoidance of talking to male clients.	11.8	
*Transgender identity's impact on condomless sex scale*		8.5 (3.4)

SD, standard deviation; CRAIMC, condomless receptive anal intercourse with male clients.

### Statistical analysis

Using CRAIMC in the last month as the dependent variable, bivariate odds ratios (ORs) of the background independent variables were estimated. Those background variables that showed *p<*0.1 in the bivariate analysis were adjusted for in subsequent multiple logistic regression analyses to derive the adjusted ORs (AORs) and respective 95% confidence intervals (CIs). Following Baron and Kenny's method [25], the first mediation hypothesis was tested by first inspecting the association between feminizing medical interventions and self-efficacy in condom use. Multiple logistic regression models, including the variables of feminizing medical interventions and self-efficacy, were then fitted. A weakened association between the variable of feminizing medical intervention and CRAIMC would suggest the presence of a mediation effect [25]. To test the second mediation hypothesis, the same procedure was repeated, but the variable of feminizing medical interventions was replaced by the Transgender Identity's Impact on Condomless Sex Scale. We used SPSS version 16.0; *p* values<0.05 were considered statistically significant.

## Results

### Background characteristics

Over half of the participants were 18 to 30 years old (50.9%), not permanent Shenyang residents (87.3%) and had stayed in Shenyang for up to five years (55.9%). Only 10.9% had attended college or university, and 41.8% had a monthly personal income >5000 RMB (806.5 USD). About 90% (91.8%) had utilized HIV prevention services in the last six months (Table 1).

Over one-quarter (26.8%) had had CRAIMC in the last month, whereas the prevalence was 32.4% among those who self-reported being HIV positive. The majority (88.6%) had had anal intercourse with non-commercial male sex partner(s) in the last month, and among these 26.2% had had condomless anal intercourse with their male sex partners (23.2% among all participants). Only 15.0% had undergone any of the following interventions: cosmetic surgery (13.2%), hormone treatment (2.3%) or sex reassignment surgery (0.5%) (Table 2).

### HIV serostatus

Prevalence of self-reported HIV-positive status was 16.8% (*n=*37). The 183 participants who self-reported HIV negative or having unknown HIV serostatus were invited to take up free HIV testing; 90 accepted the offer. The testing identified 20 HIV positive cases (22.2% among the 90 testers and 9.1% among all participants). The HIV serostatus of the 93 participants who refused to take up HIV testing remained unknown. Overall, 25.9% of all the participants either self-reported or were tested/confirmed as HIV positive (Table 1).

### Sexual practices with male clients

Almost half of the participants most often recruited their male clients at parks (44.1%) and 45% had provided sex services to male clients in a rented room. About one-third (35%) charged ≤100 RMB (≤16.1 USD) per episode of sexual practice. About two-thirds (65.4%) had had one to ten male clients (9.1% had had >30) in the last week and 4.5% had also had female clients in the last week; 22.3 and 20.9% reported having used alcohol and/or drugs, respectively, prior to or during sex with male clients in the last month. Almost half (45.5%) had performed sex work in other Chinese cities, and 3.2% had done so in other countries in the last year (Table 2).

### Perceptions related to HIV and condom use

Individual item responses and means (SD) of the scales are described in Table 3. Cronbach's alpha values of the Perceived Susceptibility for HIV Scale, Perceived Barriers against Condom Use Scale, Perceived Self-efficacy of Consistent Condom Use Scale and Transgender Identity's Impact on Condomless Sex Scale were acceptable (0.85, 0.82, 0.70 and 0.70, respectively). Single factors were identified for these four scales by using exploratory factor analysis (EFA) and explained 58.5 to 71.8% of the total variances.

### Factors associated with CRAIMC in the last month

In the bivariate analysis, only one of the background factors (education level) was significantly associated with CRAIMC, whereas two (i.e. utilization of HIV prevention services in the last six months (*p*=0.062) and unknown HIV serostatus (*p*=0.089)) were of marginal statistical significance (i.e. 0.05<*p*<0.1) (ORs are shown in Table 4). Adjusted for these three background variables, the three other variables that were significantly and positively associated with CRAIMC were 1) feminizing medical interventions (AOR: 2.22; 95% CI: 1.03, 5.11), 2) male sex clients most often recruited at hotels (AOR: 5.02; 95% CI: 1.97, 12.79) and 3) the Transgender Identity's Impact on Condomless Sex Scale score (AOR: 1.20; 95% CI: 1.09, 1.32). The two significantly and negatively associated variables were 1) charge per episode of sex practice with clients (201 to 400 RMB: AOR: 0.27; 95% CI: 0.11, 0.67; reference group: ≤100 RMB) and 2) perceived self-efficacy of consistent condom use with male clients (AOR: 0.56; 95% CI: 0.45, 0.70). Perceived susceptibility of HIV transmission was positively associated with a marginal *p*-value of 0.052 (AOR: 1.12; 95% CI: 1.00, 1.26) (Table 5).

**Table 4:** Associations between background variables and CRAIMC in the last month

	%	OR (95% CI)
Education level		
Primary or below	44.0	Reference
Junior secondary	26.6	0.46 (0.18, 1.17)
Senior secondary	22.0	0.36 (0.14, 0.91)*
Tertiary	29.2	0.52 (0.16, 1.71)
HIV prevention services utilization in the last six months		
No	44.4	Reference
Yes	25.2	0.42 (0.16, 1.13)†
HIV serostatus		
Self-reported as being HIV positive	22.0	Reference
Tested as positive in rapid HIV testing	32.4	1.92 (0.78, 4.74)
Tested as negative in rapid HIV testing	15.0	0.71 (0.18, 2.75)
Self-reported HIV negative or unknown HIV serostatus but refused to take up HIV testing	32.3	1.91 (0.92, 3.95)†

*Note*: Variables considered but that were non-significant are not listed in this table; they included age group, monthly income, resident of Shenyang and duration of stay in Shenyang.

* *p*<0.05

† *p*<0.10; OR: univariate odds ratios; CI, confidence interval. OR and 95%CI of variables with *p*<0.05 were bold.

**Table 5:** Associations between feminizing medical interventions, sexual practice with male clients, perceptions on HIV and condom use, and CRAIMC in the last month

	OR (95% CI)	AOR (95% CI)
**Feminizing medical interventions**		
Had undertaken such intervention(s)	2.71 (1.26, 5.81)*	2.22 (1.03, 5.11)*
**Sexual practice with male clients**		
Channel most often used to recruit clients		
Internet	Reference	Reference
Park	2.16 (1.07, 4.37)*	1.85 (0.86, 3.97)
Hotel	4.47 (1.84, 10.87)**	5.02 (1.97, 12.79)**
Engaged in sexual practice with clients in other Chinese cities in the last year		
No	Reference	
Yes	1.78 (0.98, 3.25)†	NS
Average charge per episode of sex with clients (in RMB)		
≤100 RMB	Reference	Reference
101 to 200 RMB	1.26 (0.54, 2.90)	1.22 (0.50, 2.98)
201 to 400 RMB	0.28 (0.12, 0.65)**	0.27 (0.11, 0.67)**
≥401 RMB	0.41 (0.17, 0.99)*	0.42 (0.16, 1.12)†
**Perceptions on HIV and condom use**		
Perceived susceptibility for HIV scale	1.13 (1.02, 1.26)*	1.12 (1.00, 1.26)†
Perceived self-efficacy in consistent condom use scale	0.54 (0.43, 0.67)***	0.56 (0.45, 0.70)***
Transgender identity's impact on condomless sex scale	1.19 (1.09, 1.31)***	1.20 (1.09, 1.32)***

*Note*: Variables that were considered but were non-significant are not listed in this table; they included anal intercourse with non-commercial male sex partner(s) in the last month, venues where sexual practices with clients took place, engaged in sexual practices with clients in other countries, number of male clients and female clients, ever used alcohol or drug prior to or during sexual practices with clients, the Perceived Barrier of Condom Use Scale and perceived cue to action of condom use.

† *p*<0.10

* *p*<0.05

** *p*<0.01

*** *p*<0.001, NS: *p>*0.10. OR, univariate odds ratios; AOR, adjusted odds ratio, odds ratios adjusted by significant background variables (educational level, HIV prevention services utilization in the last six months and HIV serostatus). OR and 95%CI of variables with *p*<0.05 were bold.

### Testing the mediation hypotheses

Perceived self-efficacy of consistent condom use with male clients was significantly correlated with the Transgender Identity's Impact on Condomless Sex Scale (Spearman *r=*−0.422, *p*<0.001). Furthermore, the significant association between this scale on perceived transgender identity's impact on condomless sex and CRAIMC became non-significant (AOR: 1.09; 95% CI: 0.98, 1.22; Table 5) after perceived self-efficacy was added to the multiple logistic regression model, with the variable of self-efficacy remaining strongly associated with CRAIMC in that model. The results suggest a full mediation effect. Feminizing medical interventions was not significantly associated with perceived self-efficacy (Spearman *r*=−0.005, *p*=0.642); therefore the second mediation hypothesis was not tested.

## Discussion

Transgender women sex workers in China are likely to be at high risk of HIV, indicated by the 25.9% HIV prevalence according to self-reported and testing data. Moreover, 40% of all participants self-reported negative or unknown HIV status and refused free HIV testing offered by this study. These participants were more likely to report CRAIMC, although this was marginally statistically significant. It is reasonable that some participants may have felt uncomfortable disclosing their HIV positive status to interviewers. Disclosure of HIV status is not a norm in China and is highly stigmatized. Therefore, the self-reported HIV prevalence was probably understated. Further research on HIV prevalence and incidence among transgender women sex workers is required to inform policymakers and health workers.

Over 25% of all participants and one-third of those who self-reported being HIV positive had engaged in CRAIMC, suggesting that we need to develop more effective and tailor-made HIV prevention interventions for transgender women sex workers. There is a dearth of NGOs serving transgender women sex workers in China. The establishment of transgender-friendly NGOs is needed. The majority of transgender women sex workers also had sex with men who were non-clients and such occasions frequently involved condomless anal intercourse. HIV interventions should take into account the different partner types for transgender women sex workers.

Importantly, 5% of the participants also served female clients. As we focused on the male clients of transgender women sex workers, we did not investigate condom use related to female clients. More research is required to understand the implications for HIV prevention. The mobility of transgender women sex workers is a concern, as this is a known risk factor for HIV transmission [26]. The majority of the participants came to Shenyang from other parts of China; moreover, half had travelled to other Chinese cities or other countries for sex work in the last year. Prevalence of CRAIMC while working in other cities was also high. Mobility may create additional challenges for designing HIV interventions for transgender women sex workers in China.

We need to understand the factors associated with CRAIMC in order to design effective HIV prevention programmes. Similar to previous studies on condomless anal intercourse among men who have sex with men (MSM) [27], education level was negatively associated with CRAIMC. Unlike studies of MSM that found 40.7 to 69.6% in Chinese cities had attained university education [28–30], our study with transgender women sex workers found that only 10.9% had a university education. The lower education level may create additional challenges for HIV prevention efforts targeting transgender women sex workers. Similar to previous studies involving female sex workers [31] and those of the two studies that surveyed transgender women sex workers [1,22], we found that a lower price for sexual practices with clients was associated with CRAIMC. About one-third of our participants charged ≤100 RMB per episode of sex. The lower income might result in financial difficulty and lower negotiation power over condom use with male clients. Attention should be given to those with low educational level and/or who charge less for their sex work.

Only 15% of study participants had taken up at least one type of feminizing medical intervention (mostly cosmetic surgeries: 13.2%), and only one had received sex reassignment surgery. It is unknown whether that participant had vaginal sex with male clients. In China, cosmetic surgeries are available at most plastic surgery hospitals nationwide. However, sex reassignment surgery is costly (about 30,000 RMB or 4838 USD) and is unaffordable for many transgender women. Accessibility is extremely low, as it can only be conducted by senior plastic surgeons of major hospitals [12], endorsement must be obtained from the applicant's next of kin, and an official proof of being free from any past criminal offenses has to be issued by local public security offices [12]. Meanwhile, sex work remains illegal in China [32]. We found that participants who underwent feminizing medical interventions were at higher risk of CRAIMC. It was not associated with self-efficacy in condom use with male clients, and its association with CRAIMC was hence not mediated by self-efficacy.

Although perceptions related to condom use, including those derived from the HBM, were associated with condomless anal intercourse in research among male sex workers [24], such factors had not been studied among transgender women sex workers. We found that self-efficacy for consistent condom use with male clients was a protective factor against CRAIMC. Concern about transgender identity's impact on condomless sex was significantly correlated with both self-efficacy and CRAIMC. Furthermore, its association with CRAIMC was fully mediated by self-efficacy. This suggests that being transgender may have created additional difficulties for condom use with male clients. The finding is consistent with results obtained from qualitative studies that found being transgender and worrying about exposure of transgender identity to male clients were obstacles to negotiating condom use with male clients [18–20]. Skills training to increase self-efficacy for condom use and address concerns about exposing one's status as a transgender woman may be important components of HIV prevention.

Another factor, perceived susceptibility for HIV transmission, was of marginal statistical significance; however, it may still be important to increase the perception of HIV transmission risk among transgender women sex workers. Perceived barriers and cue to action (e.g. display of prevention messages at sexual venues) were not significant and strategies based on these factors may not be useful for HIV prevention targeting this population in China.

Although over 90% had received HIV-related services in the last six months, prevalence of CRAIMC was high. Half of all participants did not know their HIV status, indicating much room for improvement in HIV testing and condom promotion strategies. Tailored HIV prevention services for transgender women sex workers are virtually unavailable in mainland China. They may be treated as MSM in the eyes of workers of the CDC, neglecting their unique prevention needs. Barriers for condom use, however, differ between transgender women sex workers and male sex workers. For instance, some circumstances during sex work linked to their transgender identity (e.g. worry about disclosure of transgender status) may create additional risks of CRAIMC. Needs assessments for developing tailored HIV prevention services for transgender women sex workers and their male clients are urgently warranted. Segmentation is a key to success in HIV prevention, according to social marketing principles [33]. We strongly advocate a policy review and training for CDC workers about gender identity and the special needs of transgender women sex workers.

This study has some limitations. Like previous studies [1,13,18–20,34], identity as a transgender woman was self-defined; there is no standard instrument assessing gender identity. Similar to other studies surveying hard-to-reach populations (including transgender women sex workers) [1,14–16], participants were recruited mainly through outreach and referrals. There was no sampling frame, and random sampling was not feasible. Unlike female sex workers, transgender women sex workers tend to recruit their clients *via* a relatively limited number of venues/channels (such as particular parks and Internet websites). Because we covered most of these areas when recruiting participants, we believe that our sample reflected the overall situation of transgender women sex workers in Shenyang. However, the applicability of study findings to other places in China may be limited. As face-to-face interviews were conducted, social desirability may have caused reporting biases when answering questions regarding HIV status and CRAIMC. This was a cross-sectional study that could not establish causality. In the absence of validated scales, relevant scales were constructed for this study. They demonstrated acceptable internal reliability; however, they have not been externally validated. Moreover, some potentially important factors related to CRAIMC (e.g. social support and subjective norms) were not included in this study. We did not obtain information about whether the male clients had had sex only with men, only with women or with both men and women. Lastly, we did not ask about potentially important sexual practices between transgender women sex workers and their female clients, as this study mainly focused on CRAIMC.

## Conclusions

This study, for the first time, described the complex high-risk situations of transgender women sex workers in mainland China, including the high prevalence of HIV and CRAIMC, high intercity mobility involving sex work and involvement in sexual networks consisting of multiple types of sex partners. Interventions are urgently needed and should target transgender women sex workers with lower education levels and those who charge less for sex work services. Factors unique to transgender women sex workers further increased the risk of CRAIMC, such as feminizing medical intervention and perceived transgender identity's impact on condomless sex. Skills training should be provided to increase self-efficacy in condom use. Further research is warranted to better understand the determinants of CRAIMC and to develop effective intervention programmes.
